# Statin-Induced Necrotizing Autoimmune Myositis: Diagnosis and Management

**DOI:** 10.7759/cureus.13787

**Published:** 2021-03-09

**Authors:** David Cha, Fan Wang, Basanti Mukerji, Vaskar Mukerji

**Affiliations:** 1 Internal Medicine, Wright State University Boonshoft School of Medicine, Dayton, USA; 2 Rheumatology, Dayton VA Medical Center, Dayton, USA; 3 Rheumatology, Wright State University Boonshoft School of Medicine, Dayton, USA; 4 Cardiology, Dayton VA Medical Center, Dayton, USA; 5 Cardiology, Wright State University Boonshoft School of Medicine, Dayton, USA; 6 Cardiology, Kettering Medical Center, Dayton, USA

**Keywords:** necrotizing myositis, statin-induced necrotizing autoimmune myopathy, anti-hmgcr, immune modulation therapy

## Abstract

Statins are among the most frequently prescribed drugs as they effectively lower cardiovascular mortality. Atherosclerotic plaques are stabilized and lipid levels are lowered, as statins inhibit 3-hydroxy-3-methylglutaryl coenzyme A (HMG-CoA) reductase. Patients placed on these drugs frequently report muscle aches, but true myositis that would call for discontinuance of the drug is actually uncommon. Workup for statin-induced myositis would require ruling out other causes of myositis and muscular dystrophies, and this can often be perplexing for the primary care physician to whom these patients initially present. This case report and recommendations may serve as a helpful guide.

## Introduction

Myalgia is the most commonly reported adverse symptom when statins are prescribed [1-4]. The frequency may be as high as 30% with the onset being within weeks of starting therapy or several years later [5]. Many patients choose to discontinue therapy. Yet, studies suggest that patients who stop taking a statin due to myalgia can tolerate it later when re-prescribed [6,7]. Furthermore, the reported frequency of myalgia in clinical practice is higher than what has been reported in clinical trials [7]. The recently concluded SAMSON (Self-Assessment Method for Statin Side-Effects or Nocebo) trial showed that patients on statins do get side effects, but 90% of this symptom burden can occur with a placebo tablet as well. Hence, this was a nocebo effect [8]. For individuals treated with statins, the estimated frequency of muscle damage with weakness and elevated levels of creatinine kinase (CK) is approximately 1 in 10,000 [9]. In most cases, patients recover spontaneously when the statin is discontinued. In very rare cases, an autoimmune myositis develops in patients on statins. This is associated with the presence of IgG (immunoglobulin G) autoantibodies against 3-hydroxy-3-methylglutaryl coenzyme A (HMG-CoA) reductase, with muscle-cell necrosis on biopsy with regenerating cells [10]. Accurate diagnosis is crucial for the treatment of this condition. We present here a case of statin-induced necrotizing autoimmune myositis (NAM) and provide information on its diagnosis and management.

## Case presentation

**Figure 1:**
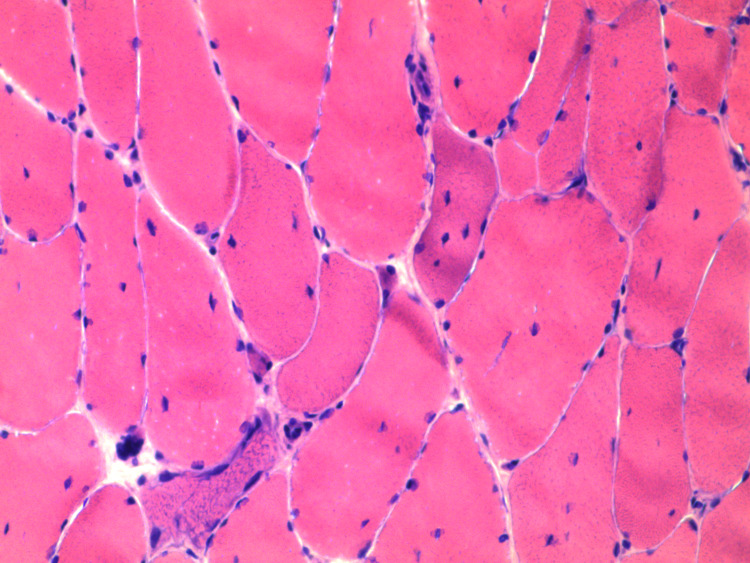
H&E stain of the muscle biopsy sample from right vastus lateralis. This figure showed marked fiber size variation with degenerating and regenerating muscle cells with no inflammation. H&E, hematoxylin and eosin

**Figure 2:**
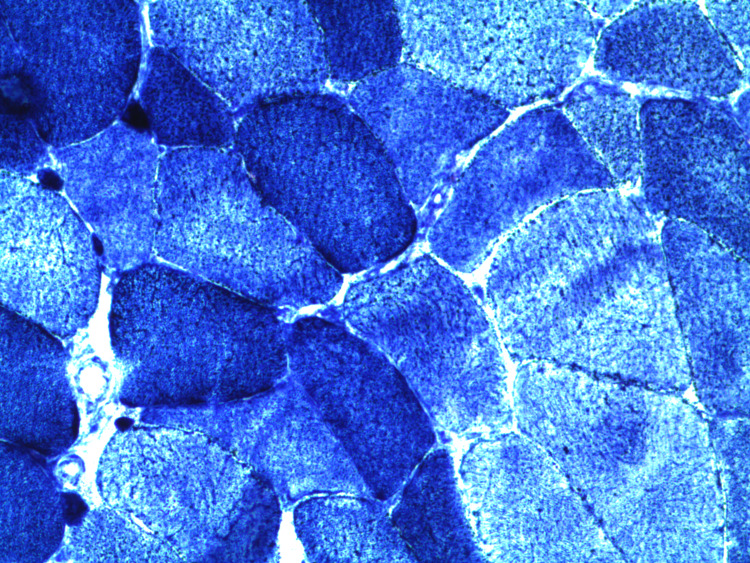
NADH stain showed that some muscle fibers had an increased uptake of HMG-CoA reductase enzyme. NADH, Nicotinamide adenine dinucleotide hydrogenase; HMG-CoA; 3-hydroxy-3-methylglutaryl coenzyme A

**Figure 3:**
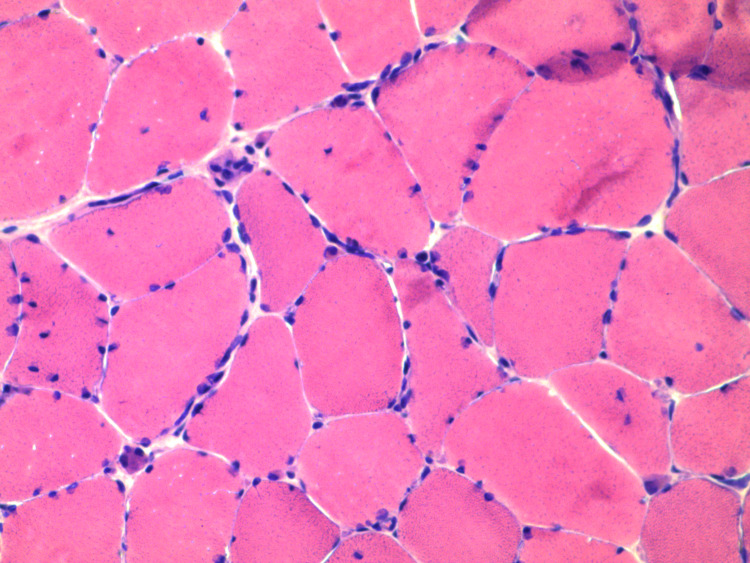
H&E stain of the muscle biopsy sample from the right vastus lateralis. This imaging showed macrophage clusters in between the muscle cells, which may help in tissue repair. Many of the muscle cells were nucleated, suggesting damage to the cell. H&E, hematoxylin and eosin

A 57-year-old man with a past medical history of hyperlipidemia and hypertension presented with six months of progressive bilateral proximal arm and leg muscle weakness accompanied by fatigue. He had been on atorvastatin 40 mg daily for three years prior to the onset of his symptoms, and it was stopped by his primary care physician (PCP). He had no history of any recent infection, travel, radiation, surgeries, or any vaccinations. He was receiving metoprolol succinate 25 mg daily. He denied using any over-the-counter medications. The patient had significant difficulty getting out of his chair. There was no focal neurological deficit. Examination of his muscles revealed that his proximal muscles in upper and lower extremities had 4/5 muscle strength. Distal muscle strength in both upper and lower extremities was 5/5. The rest of his physical examination was essentially normal. Laboratory studies revealed normal complete blood count and electrolyte levels. The thyroid-stimulating hormone (TSH) level was normal. His CK was elevated at 8,121 units/liter. Antinuclear antibody (ANA) was negative as were anti-synthetase antibodies and anti-signal recognition particle (SRP). A drug screen was performed to rule out cocaine- or alcohol-induced myositis, and it was negative. A whole-body positron emission tomography (PET) scan was negative ruling out a paraneoplastic cause for NAM. An electromyography (EMG) was performed, which showed changes consistent with myositis in the proximal muscles of his legs. HMG-CoA reductase antibody was positive at 149 (normal value: 0-19; strongly positive: >60). He had a muscle biopsy of his right vastus lateralis, as seen in Figures 1-3.

The patient was started on oral prednisone 60 mg daily and mycophenolate mofetil 500 mg twice a day. He received physical therapy and was given calcium and vitamin D3 supplements for the prevention of steroid-induced osteoporosis. After three months of this treatment, his muscular weakness began to resolve. The patient came for serial clinic visits to measure muscle strength and CK level, which showed slow normalization through the next few months. A year later, the patient returned to the clinic alone without his wife’s assistance for the first time. His muscle strength had almost returned to normal. The prednisone was tapered off in six months, and mycophenolate mofetil was tapered off after 14 months. The patient’s CK had returned to normal at 43 and remained normal during subsequent visits.

## Discussion

The workup of patients on statins reporting muscle weakness and pain can be challenging for any PCP. As the SAMSON study demonstrated, many patients who complain of muscle pain with statins may actually be experiencing a nocebo effect [8]. Others may have myositis. It is important in the evaluation of patients to start with a detailed history. Important elements of the history are recent infections, travel, other medications, substance abuse, and vaccination. Certain viruses, bacteria, parasites, and medications can cause symptoms that mimic myositis [2-4,10,11]. A family history is helpful to rule out muscular dystrophies and metabolic myopathies [11]. Physical examination identifies the affected muscle groups. The clinical presentation for Anti-HMG-CoA reductase associated NAM typically includes progressive bilateral proximal upper and lower limb weakness causing difficulty in rising from a low chair, combing hair, and climbing steps that persist despite discontinuing statins [3,10].

**Figure 4:**
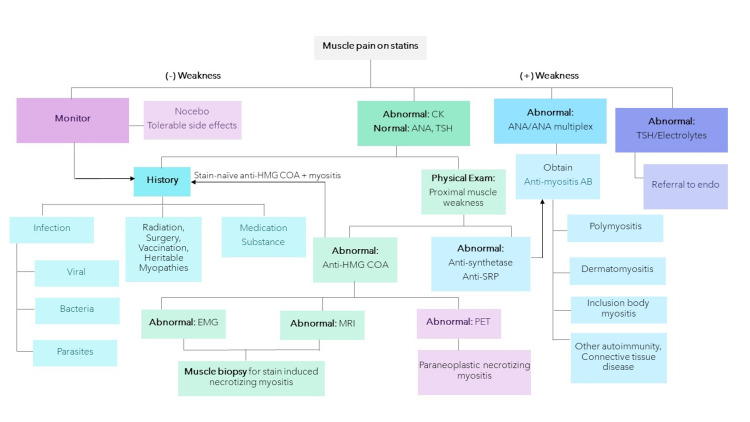
Evaluation of statin-associated muscle pain and weakness. CK, creatinine kinase; ANA, antinuclear antibody; TSH, thyroid-stimulating hormone; AB, antibodies; HMG-CoA, 3-hydroxy-3-methylglutaryl coenzyme A; SRP, signal recognition particle; EMG, electromyography; PET, positron emission tomography

Initial laboratory workup should include checking for CK levels and ANA. Patients with NAM have CK levels typically between 1,000 and 10,000 units/liter and negative ANA testing [2-4]. In NAM, further lab testing such as anti-myositis antibodies (anti-synthetase antibodies, anti-SRP antibodies) and anti-HMG-CoA reductase can be ordered [2-4]. Recent information shows that the anti-HMG-CoA reductase antibody is a powerful tool for diagnosis, with a sensitivity of 99.4% and specificity of 99.3% [11-14]. Negative ANA, anti-synthetase antibodies, and anti-SRP antibodies help rule out other inflammatory myositis such as dermatomyositis, polymyositis, and anti-synthetase syndromes [14]. EMG, MRI, and muscle biopsy may be considered, as shown in Figure 4. Patients with true NAM have EMG changes that show myopathic motor-unit potentials consisting of short-duration, low-amplitude polyphasic units on voluntary activation and increased spontaneous activity with fibrillations, complex repetitive discharges, and positive sharp waves [14,15]. Muscle biopsy shows necrotizing myositis, with no inflammatory infiltrates and only macrophages invading necrotic and regenerating muscle fibers. The biopsy finding separates this disease from other inflammatory myositis [15,16]. MRI shows T1 hyperintensity in affected muscles [14]. A whole-body PET scan can rule out a paraneoplastic cause for NAM [14,17].

The initial treatment of anti-HMG-CoA reductase associated NAM is to stop the statin. If the patient’s symptoms do not improve and continue to progress, steroids such as prednisone or methylprednisolone are the next line of treatment [14,18]. Initially prednisone is used at 1 mg per kilogram of body weight per day with a maximum of 80 mg daily. If high-dose prednisone fails to improve the patient’s symptoms or decrease the CK levels, immunosuppressive therapies can be added. Methotrexate, azathioprine, and mycophenolate mofetil have all been used. For those who have moderate symptoms, immunosuppressive medications can be used as initial therapy in combination with prednisone. If patients do not respond to combination therapy after 8 to 12 weeks, intravenous immunoglobulin (IVIG) or rituximab may be added. In severe cases, IVIG can be considered as the first line of treatment. The duration of treatment is determined by the patient’s symptoms and the normalization of CK. Rarely patients have long-term symptoms of falling and muscle weakness and this could be due to permanent muscle injury and replacement of muscle tissue with fat as in polymyositis and dermatomyositis. This can be investigated with an MRI [14,18]. Exercise can be a helpful adjunctive therapy [12,14].

## Conclusions

NAM is an uncommon complication of statin use that requires discontinuance of the drug. It was first reported as a histopathological entity in the early 2000s. In the differential diagnosis, other causes of muscular weakness need to be ruled out, including muscular dystrophies, inflammatory myositis, and paraneoplastic syndromes. In all cases, anti-synthetase antibodies, anti-SRP antibodies, and HMG-CoA reductase antibodies should be checked, as their presence would point toward the cause for the NAM.
